# A *Haemophilus influenzae*-associated mycotic aneurysm months after treated bacteremia

**DOI:** 10.1128/asmcr.00027-25

**Published:** 2025-06-12

**Authors:** Josiah Bennett, Sarah Lohsen, Elizabeth Y. Kim, Pranavi Ravichandran, William Blake, Kimberly A. Workowski, Sarah W. Satola, Matthew H. Collins, Danielle B. Steed

**Affiliations:** 1 Department of Medicine, Emory University School of Medicine 234195, Atlanta, Georgia, USA; 2 Division of Infectious Diseases, Department of Medicine, Emory University School of Medicine 234195, Atlanta, Georgia, USA; 3 Division of Vascular Surgery, Department of Surgery, Emory University School of Medicine 160352, Atlanta, Georgia, USA; 4 Georgia Emerging Infections Program, Atlanta, Georgia, USA; Vanderbilt University Medical Center, Nashville, Tennessee, USA

**Keywords:** nontypeable *Haemophilus influenzae*, mycotic aneurysm, invasive infection, HIV

## Abstract

**Background:**

Invasive nontypeable *Haemophilus influenzae* (NTHi) infections increased dramatically in the Atlanta area in 2017–2018. Most isolates from these infections belonged to two closely related genetic lineages and were likely being spread through sexual networks of men living with HIV.

**Case Summary:**

We present a case of a 63-year-old male with well-controlled HIV and treated NTHi pericarditis and bacteremia who we diagnosed with an infected right superficial femoral artery pseudoaneurysm due to NTHi. The causative strain belonged to one of the lineages identified in the 2017–2018 case clusters.

**Conclusion:**

This case illustrates that novel disease phenotypes continue to emerge for unusually pathogenic strains of NTHi.

## INTRODUCTION

*Haemophilus influenzae* is a gram-negative coccobacillus to pleomorphic rod that commonly colonizes the upper respiratory tract of healthy humans, who are the bacterium’s only known natural reservoir. This species is subdivided into seven groups: six that express distinct forms of the polysaccharide capsule, known as serotypes a through f, and one unencapsulated group designated nontypeable *H. influenzae* (NTHi). While NTHi most frequently causes otitis media, sinusitis, and exacerbations of chronic obstructive pulmonary disease, it can also cause invasive disease (1, 2). Since the introduction of the *H. influenzae* serotype b vaccine, there has been a phenomenon of strain replacement whereby NTHi is now the leading cause of invasive disease across all age groups, but with a predilection for those at the extremes of age (<1 and ≥65 years old) (3, 4). Predisposing medical conditions for invasive infection include chronic lung disease, malignancy, pregnancy, and poorly controlled HIV infection (4–9). The most commonly reported invasive NTHi clinical syndromes are bacteremia, with or without pneumonia, and meningitis (4, 5). NTHi is typically genetically diverse, and clusters of infection are uncommon. However, there was an increase in invasive NTHi infection observed among men living with HIV in metropolitan Atlanta from 2017 to 2018, with septic arthritis being a predominant disease manifestation (10). Most isolates from these infections belonged to two closely related genetic lineages and were likely being spread through social networks. The full spectrum of manifestations of these novel NTHi lineages remains obscure. The case described here illustrates pericardial and vascular manifestations of invasive NTHi that are extremely uncommon. Preliminary genotyping indicates the causative strain belongs to one of the lineages identified in the 2017–2018 case clusters. Thus, novel phenotypes of disease continue to emerge for unusually pathogenic strains of NTHi circulating in Atlanta.

## CASE PRESENTATION

A 63-year-old man presented with a 5-day history of progressive right proximal thigh swelling and pain, which limited ambulation. He had a history of well-controlled HIV, hypertension, and hyperlipidemia. He denied tobacco, alcohol, and recreational drug use, including IV drug use. For his sexual history, he reported having sex with men only. His medications included the combination of zidovudine-lamivudine-abacavir, fosamprenavir, and ritonavir.

Three months prior to this admission, he was admitted to the same hospital with a large pericardial effusion that was complicated by cardiac tamponade, for which he underwent pericardiocentesis. NTHi grew in two out of two blood culture sets and pericardial fluid cultures. The time to positivity for his blood cultures was 21 hours. This prior presentation was characterized by chills, shortness of breath, and arthralgias. His hospitalization was complicated by atrial fibrillation with rapid ventricular rate and a right gastrocnemius deep vein thrombosis, for which he continued anticoagulation. He also presented with ongoing arthralgias affecting bilateral knees and ankles, which was managed as reactive arthritis in consultation with rheumatology. Intracardiac infection was excluded by transthoracic and transesophageal echocardiography. Two sets of repeat blood cultures showed no bacterial growth, and the patient completed 4 weeks of intravenous (IV) ceftriaxone therapy.

**Fig 1:**
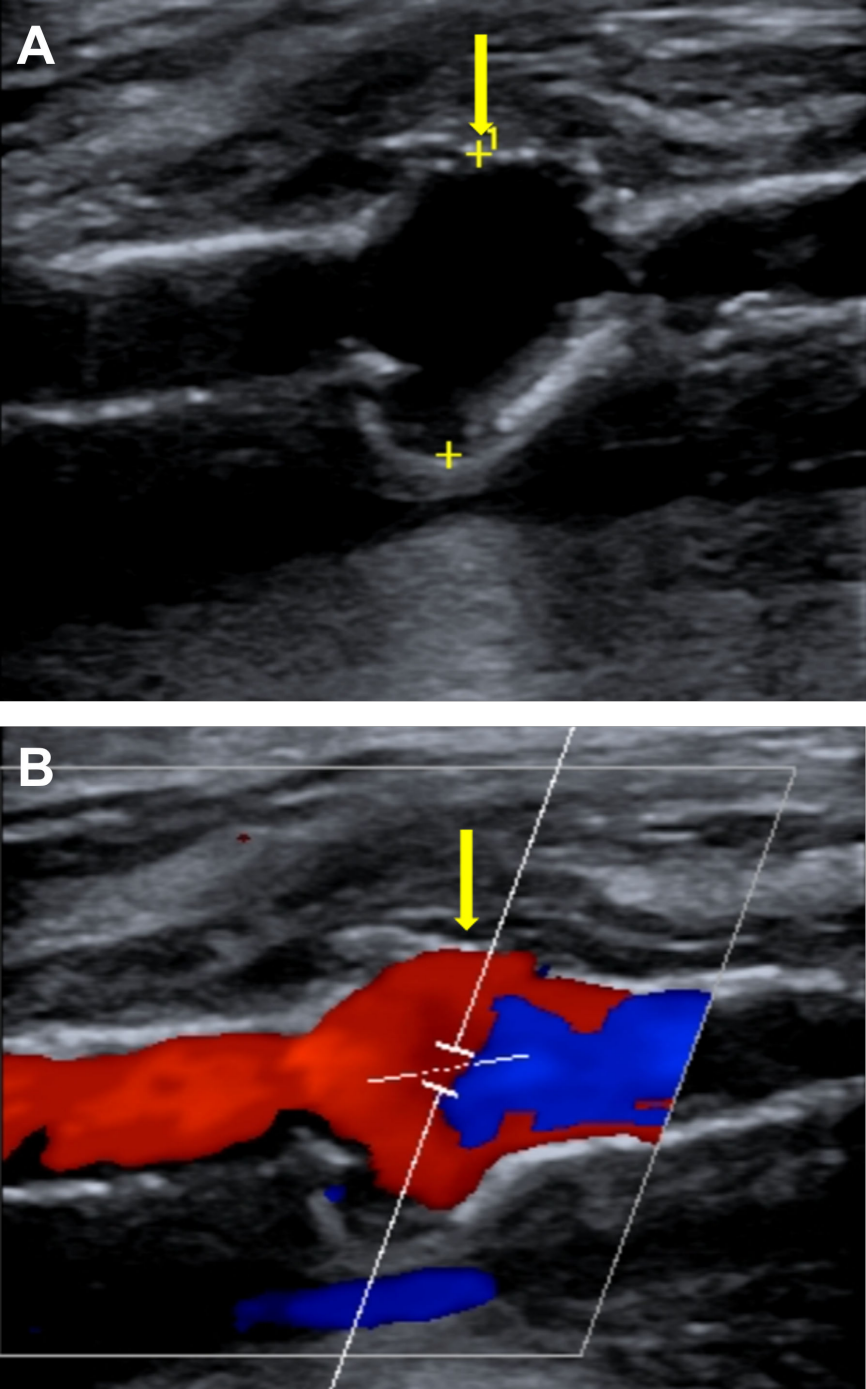
Ultrasound of the right superficial femoral artery. The images show representative longitudinal views of the vessel (**A**) without Doppler and (**B**) with Doppler. Yellow arrows indicate the pseudoaneurysm.

**Fig 2:**
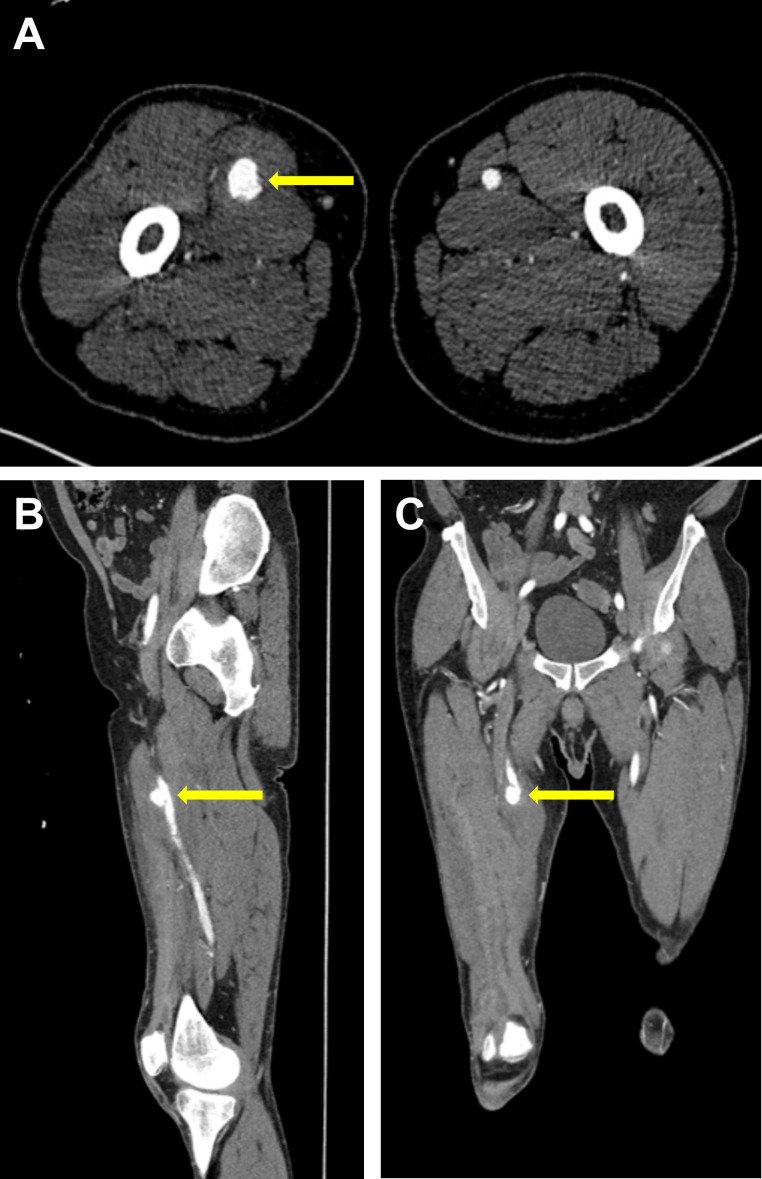
The patient’s initial computed tomographic scan showing the pseudoaneurysm of the right superficial femoral artery (yellow arrows) on representative axial (**A**), sagittal (**B**), and coronal (**C**) views.

On this admission, he was afebrile (T 36.5°C), well-appearing, but with a tender, pulsatile fullness in the right upper medial thigh without overlying skin changes or limb edema. The remainder of his exam was within normal limits. Laboratory examination revealed mild thrombocytosis (471 × 10^3^/mcL [reference range 150−400 × 10^3^/mcL]) but was otherwise unremarkable. The patient’s CD4^+^ T cell count was 394 cells per μL and CD4^+^ T cell percentage was 21.8% (reference ranges 404–1612 cells per μL and 33.0–58.0%). His HIV viral load was 26 copies per mL (reference range ≤20 copies per mL). Right lower extremity ultrasound revealed a new focal saccular aneurysmal dilatation of the proximal superficial femoral artery (SFA) suspicious for pseudoaneurysm that had not been present on ultrasound imaging during his prior hospitalization (Fig. 1). Computed tomography angiography (Fig. 2) revealed a pseudoaneurysm of the right SFA that corresponded to the patient’s symptoms as well as mild atherosclerotic calcifications involving the infrapopliteal vessels. Vascular surgery was consulted, and the patient’s findings were felt to be suspicious for a mycotic aneurysm, defined as arterial wall dilation secondary to infection. Therefore, open surgical repair via aneurysm resection and autogenous saphenous vein bypass construction was favored over percutaneous stent graft repair to mitigate the risk of retained infection. The infectious diseases team was consulted and recommended perioperative ceftriaxone given the history of recent NTHi bacteremia. No purulence was encountered upon direct exploration of the right SFA pseudoaneurysm. However, there was a prohibitive amount of soft tissue inflammation and scarring that precluded arterial dissection and excision, so he underwent right SFA bypass with reversed saphenous vein and interval ligation to exclude the pseudoaneurysmal segment. A sample of adherent perivascular soft tissue and a swab of the wound bed were sent for microbiological cultures.

His admission blood cultures, drawn prior to the initiation of antibiotics, turned positive for gram-negative bacilli in one of two sets. The time to positivity was 17 hours. Greenish-gray colonies were seen on chocolate agar. The isolate was identified as *H. influenzae* by Matrix-Assisted Laser Desorption/Ionization Time-of-Flight. The β-lactamase/cefinase test was performed and was negative. The perivascular soft tissue culture showed no bacterial growth, and the wound bed swab had delayed growth (>72 hours) of one colony of *Staphylococcus lugdunensis*, deemed a contaminant given the low organismal burden and a more likely microbiological diagnosis. The *H. influenzae* isolates from his prior hospitalization and this hospitalization were processed through the Active Bacterial Core surveillance (11), a component of the Georgia Emerging Infections Program (EIP), funded by the Centers for Disease Control and Prevention (CDC) (7) and the Emory University Investigational Clinical Microbiology Core (https://www.cores.emory.edu/icmc/). The isolates were sent to the Georgia Department of Public Health laboratory where they were serotyped by rtPCR and found to be unencapsulated or nontypeable. For whole-genome sequencing (WGS) analysis, bacterial DNA was extracted using the Zymo Quick-DNA Fungal/Bacterial Miniprep Kit (Zymo Research, Irvine, CA), and Illumina WGS was conducted by SeqCenter, LLC (Pittsburgh, PA). Using Bactopia (12), raw sequence reads were filtered for quality, and the multilocus sequence types were determined (*H. influenzae* PubMLST scheme; https://pubmlst.org/hinfluenzae/) (13). The Bacterial and Viral Bioinformatics Resource Center (BV-BRC) (14) was used to query variation in isolate genomes using the Variation Analysis Service. Average nucleotide identity (ANI) was calculated using the ANI Calculator (15). WGS and phylogenetic analysis of this admission’s blood *H. influenzae* isolate indicated it belonged to the same strain as that which caused his earlier episode of bacteremia and pericarditis (ANI of 99.99%), and he was subsequently diagnosed with recurrent NTHi bacteremia and mycotic aneurysm due to this organism. Repeat transthoracic and transesophageal echocardiograms showed no abnormalities, and follow-up postoperative blood cultures, drawn after 4 days of ceftriaxone, showed no growth. The patient was discharged and completed 6 weeks of IV ceftriaxone 2 g every 24 hours plus an additional 2 weeks of oral cefdinir 300 mg twice daily. At 15 months of post-treatment follow-up, he was doing well without right lower extremity pain or dysfunction. Serial ultrasounds have demonstrated successful exclusion of the prior SFA pseudoaneurysm with a widely patent bypass graft. He has remained without radiographic or clinical signs of infection.

## DISCUSSION

The emergence of novel invasive NTHi infections in Atlanta, Georgia, with unusual disease features, particularly among persons living with HIV, was observed in 2017–2018. In the study evaluating the epidemiological, clinical, and genetic features of this outbreak (10), a majority of the isolates from persons living with HIV were identified as clonal, belonging to two genetically distinct but closely related NTHi lineages. Geospatial analysis also showed that the infections caused by these two clonal groups clustered in close geographic proximity, suggesting transmission within a social network, possibly through sexual contact. While the primary route of *H. influenzae* transmission is through exposure from respiratory droplets, *Haemophilus* spp. have also been cultured in patients with non-gonococcal urethritis, and orogenital contact has been suspected as an alternative mode of transmission (16–18). This underscores the pertinence of obtaining a thorough sexual history in the evaluation of patients with *Haemophilus* spp. infections. Persons with HIV who had these clonal isolates were more likely to be men who have sex with men, more likely to have a history of sexually transmitted illnesses, and more likely to have HIV virologic suppression, which was in stark contrast to the prevailing paradigm that invasive NTHi infection generally occurs in persons with HIV that is poorly controlled (6–9). Septic arthritis was also the most commonly reported clinical syndrome, an atypical manifestation of invasive NTHi in adults (19). Notably, this patient had arthralgias and knee effusions as part of his initial presentation, which raised suspicion for septic arthritis. However, no bacteria were recovered from the synovial fluid with the caveat that he had been on antibiotics in the week prior to arthrocentesis, potentially sterilizing these cultures.

This case further highlights pericardial and vascular manifestations of invasive NTHi that are extremely uncommon. The majority of *H. influenzae* adult purulent pericarditis cases are attributable to serotype b (20). In our review of the literature, we identified only two adult cases caused by NTHi, and in both—one in an immunosuppressed female with rheumatoid arthritis and the other in an immunocompetent female with mild asthma—the development of purulent pericarditis was preceded by a pulmonary source of infection (20, 21), which was not the case with our patient.

Similarly, *H. influenzae* is very rarely implicated in mycotic aneurysms. We found 17 reports in the literature (22–28), the majority of which involved the aorta. Most of these patients had predisposing conditions, such as older age, smoking, respiratory disease, and other immunocompromising conditions. Sources of infection varied but included upper respiratory tract infection, microbial arteritis, and vertebral osteomyelitis. In the cases in which typing was accomplished or specified, only encapsulated strains of *H. influenzae* were identified. Mycotic aneurysms can be difficult to diagnose as their presentations can be protean depending on the site of aneurysmal involvement, and diagnosis relies on a combination of clinical presentation, laboratory, radiological, and intraoperative findings. Blood culture positivity varies from 20 to 90% depending on the mycotic aneurysm site (29). Given the mechanisms by which infected aneurysms are thought to arise (septic embolization, contiguous extension, hematogenous infection during bacteremia, direct blood vessel contamination, or trauma [29]), a high index of suspicion should be maintained in patients with a corresponding history, such as recent bacteremia in this case. While we were unable to obtain a sample of the affected artery wall for microbiological testing, positive blood cultures, suggestive radiographic morphology, the absence of any preceding trauma or iatrogenic cause for arterial wall injury at this site, and intraoperative findings of extensive inflammation supported a diagnosis of mycotic SFA aneurysm secondary to *H. influenzae*.

Genotyping indicated that the causative strain in this case belongs to one of the clonal lineages identified in the 2017–2018 Atlanta case clusters. As the WGS comparison of both of his isolates indicates an ANI of 99.99% (which exceeds the 99.9% ANI threshold at which two strains are typically considered identical), the isolates that caused his earlier episode of bacteremia and pericarditis and this episode of mycotic aneurysm are the same strain. However, it remains unresolved whether this patient sustained the relapsed infection due to reinfection with the same isolate (i.e., from continued contact with a colonized partner) versus recrudescence due to bacterial persistence. Our genomic analysis may favor the latter mechanism. A comparison of the isolates showed 44 changes, with predicted high-impact protein-level effects for genes involved in N-linked glycosylation, sialylation, lipooligosaccharide (LOS) biosynthesis, thymidine metabolism, and iron transport (Table 1). Changes in some of the genes involved in these biosynthetic pathways have been previously implicated in enhanced virulence, persistence, and immune evasion. Like other gram-negative organisms, *H. influenzae* is susceptible to complement-mediated cytolytic activity. Evasion and/or survival of complement-mediated attack is essential for pathogenesis. Since NTHi strains do not possess a capsule, their resistance to human complement relies on other mechanisms, such as LOS modification. LOS contributes to *H. influenzae* survival; for example, sialylation has been shown to protect epitopes on NTHi LOS from being targeted by bactericidal IgM (30). Many LOS biosynthetic genes undergo rapid on/off switching of expression, known as phase variation, which seems to be important for pathogenicity and has been associated with the transition from nasopharyngeal colonization to middle ear invasion during otitis media (31, 32). Iron homeostasis is essential in many bacteria, including NTHi, and the careful regulation of iron uptake in certain host microenvironments may be important for NTHi persistence (33).

**TABLE 1:** Whole genome sequencing comparison of the patient’s NTHi isolates^*a*^

Contig	Pos	Score	Var_cov	Var_frac	Type	Ref_nt_pos_change	Ref_aa_pos_change	Gene_ID	Function	snpEff_type	snpEff_impact
727.3669.con.0001	39485	533.178	27	0.64	Insertion	184_187dupGCAA	Lys63fs	fig|727.3669.peg.33	Phage protein	frameshift_variant	HIGH
727.3669.con.0001	520297	2462.64	87	0.88	Deletion					intergenic_region	MODIFIER
727.3669.con.0002	259202	6504.25	209	1	Deletion	242delT	Val81fs	fig|727.3669.peg.845	Thymidine kinase (EC 2.7.1.21)	frameshift_variant	HIGH
727.3669.con.0002	276820	2936.6	96	0.98	Deletion	36_43delCGAGCATA	Glu13fs	fig|727.3669.peg.867	Beta-1,4-galactosyltransferase	frameshift_variant	HIGH
727.3669.con.0002	279943	2378.79	97	0.88	Deletion	119_122delATCA	Asn60fs	fig|727.3669.peg.871	Lipooligosaccharide biosynthesis protein lex-1 (EC 2.-.-.-)	frameshift_variant	HIGH
727.3669.con.0002	52060	854.046	36	0.62	Insertion	3_4insTCAA	Lys31fs	fig|727.3669.peg.617	CMP-N-acetylneuraminate-beta-galactosamide-alpha-2,3-sialyltransferase (EC 2.4.99.-)	frameshift_variant	HIGH
727.3669.con.0005	55659	2565.28	93	0.88	Insertion	3_4insACAG	Met14fs	fig|727.3669.peg.1317	Glycosyl transferase	frameshift_variant	HIGH
727.3669.con.0007	10	343.467	19	0.83	Insertion	74_75insCCAA	Asn51fs	fig|727.3669.peg.1448	Outer membrane receptor proteins, mostly Fe transport	frameshift_variant	HIGH
727.3669.con.0011	27437	705.276	276	0.58				fig|727.3669.rna.37	5S rRNA ## 5S ribosomal RNA	intergenic_region	MODIFIER
727.3669.con.0011	27471	502.404	216	0.55				fig|727.3669.rna.37	5S rRNA ## 5S ribosomal RNA	intergenic_region	MODIFIER
727.3669.con.0013	50	2865	223	0.63	Nonsyn	50T > C	Val17Ala	fig|727.3669.peg.1746	Glucosamine-6-phosphate deaminase (EC 3.5.99.6)	missense_variant	MODERATE
727.3669.con.0013	6	667.536	117	0.53	Nonsyn	6C > A	Asn2Lys	fig|727.3669.peg.1746	Glucosamine-6-phosphate deaminase (EC 3.5.99.6)	missense_variant	MODERATE
727.3669.con.0017	3356	260.4	11	1	Synon	3G > A	Val1Val	fig|727.3669.peg.1809	Translation elongation factor Tu	synonymous_variant	LOW
727.3669.con.0020	1970	204.009	142	0.52	Synon	12_15delGACTinsAACG	6	fig|727.3669.peg.1816	Lipopolysaccharide cholinephosphotransferase LicD1 (EC 2.7.8.-)	synonymous_variant	LOW
727.3669.con.0020	2001	469.911	159	0.52	Nonsyn	43G > A	Asp15Asn	fig|727.3669.peg.1816	Lipopolysaccharide cholinephosphotransferase LicD1 (EC 2.7.8.-)	missense_variant	MODERATE
727.3669.con.0020	2006	679.361	165	0.53	Synon	48C > A	Ile16Ile	fig|727.3669.peg.1816	Lipopolysaccharide cholinephosphotransferase LicD1 (EC 2.7.8.-)	synonymous_variant	LOW
727.3669.con.0020	2034	121.991	127	0.5	Nonsyn	76_77delAAinsCG	Lys26Arg	fig|727.3669.peg.1816	Lipopolysaccharide cholinephosphotransferase LicD1 (EC 2.7.8.-)	missense_variant	MODERATE
727.3669.con.0020	2138	782.811	99	0.58	Synon	180C > T	Arg60Arg	fig|727.3669.peg.1816	Lipopolysaccharide cholinephosphotransferase LicD1 (EC 2.7.8.-)	synonymous_variant	LOW
727.3669.con.0020	2152	76.1225	78	0.5	Nonsyn	194_198delAATTTinsGGTTC	Lys65Arg	fig|727.3669.peg.1816	Lipopolysaccharide cholinephosphotransferase LicD1 (EC 2.7.8.-)	missense_variant	MODERATE
727.3669.con.0020	2198	558.303	104	0.54	Nonsyn	240G > A	Met80Ile	fig|727.3669.peg.1816	Lipopolysaccharide cholinephosphotransferase LicD1 (EC 2.7.8.-)	missense_variant	MODERATE
727.3669.con.0020	2204	109.267	89	0.5	Synon	246_249delAGTAinsGGTG	84	fig|727.3669.peg.1816	Lipopolysaccharide cholinephosphotransferase LicD1 (EC 2.7.8.-)	synonymous_variant	LOW
727.3669.con.0020	2213	343.117	93	0.53	Synon	255_258delCATAinsTATC	87	fig|727.3669.peg.1816	Lipopolysaccharide cholinephosphotransferase LicD1 (EC 2.7.8.-)	synonymous_variant	LOW
727.3669.con.0020	2280	472.993	78	0.54	Nonsyn	322_324delAAAinsGCT	Lys108Ala	fig|727.3669.peg.1816	Lipopolysaccharide cholinephosphotransferase LicD1 (EC 2.7.8.-)	missense_variant	MODERATE
727.3669.con.0020	2294	586.477	81	0.55	Synon	336C > T	Ser112Ser	fig|727.3669.peg.1816	Lipopolysaccharide cholinephosphotransferase LicD1 (EC 2.7.8.-)	synonymous_variant	LOW
727.3669.con.0020	2337	707.574	72	0.6	Nonsyn	379A > G	Asn127Asp	fig|727.3669.peg.1816	Lipopolysaccharide cholinephosphotransferase LicD1 (EC 2.7.8.-)	missense_variant	MODERATE
727.3669.con.0020	2345	417.887	16	1	Nonsyn	387T > G	Asn129Lys	fig|727.3669.peg.1816	Lipopolysaccharide cholinephosphotransferase LicD1 (EC 2.7.8.-)	missense_variant& splice_region_variant	MODERATE
727.3669.con.0022	363	670.871	276	0.52	Synon	363A > G	Val121Val	fig|727.3669.peg.1817	Translation elongation factor Tu	synonymous_variant	LOW
727.3669.con.0022	375	1179.71	298	0.53	Synon	375T > A	Leu125Leu	fig|727.3669.peg.1817	Translation elongation factor Tu	synonymous_variant	LOW
727.3669.con.0022	402	672.496	260	0.52	Synon	402_405delCACGinsTACA	136	fig|727.3669.peg.1817	Translation elongation factor Tu	synonymous_variant	LOW
727.3669.con.0022	546	130.04	263	0.5	Synon	546T > C	Ile182Ile	fig|727.3669.peg.1817	Translation elongation factor Tu	synonymous_variant	LOW
727.3669.con.0026	203	157.887	110	0.51	Synon	201_207delTGGTGTAinsAGGGGTT	70	fig|727.3669.peg.1821	Outer membrane receptor proteins, mostly Fe transport	synonymous_variant	LOW
727.3669.con.0026	260	1054.49	167	0.57	Synon	258T > C	Thr86Thr	fig|727.3669.peg.1821	Outer membrane receptor proteins, mostly Fe transport	synonymous_variant	LOW
727.3669.con.0026	285	1240.21	165	0.55	Synon	283C > T	Leu95Leu	fig|727.3669.peg.1821	Outer membrane receptor proteins, mostly Fe transport	synonymous_variant	LOW
727.3669.con.0026	447	368.093	103	0.53	Nonsyn	445C > A	Leu149Ile	fig|727.3669.peg.1821	Outer membrane receptor proteins, mostly Fe transport	missense_variant	MODERATE
727.3669.con.0028	159	588.687	75	0.56	Nonsyn	282_289delTCACATTGinsCCATGTTA	IleVal96ValIle	fig|727.3669.peg.1822	Lipopolysaccharide cholinephosphotransferase LicD1 (EC 2.7.8.-)	missense_variant	MODERATE
727.3669.con.0028	198	678.98	86	0.57	Nonsyn	250A > C	Lys84Gln	fig|727.3669.peg.1822	Lipopolysaccharide cholinephosphotransferase LicD1 (EC 2.7.8.-)	missense_variant	MODERATE
727.3669.con.0028	208	646.173	86	0.57	Nonsyn	239_240delGTinsAC	Cys80Tyr	fig|727.3669.peg.1822	Lipopolysaccharide cholinephosphotransferase LicD1 (EC 2.7.8.-)	missense_variant	MODERATE
727.3669.con.0028	235	888.265	89	0.6	Synon	213C > T	Gly71Gly	fig|727.3669.peg.1822	Lipopolysaccharide cholinephosphotransferase LicD1 (EC 2.7.8.-)	synonymous_variant	LOW
727.3669.con.0028	255	722.501	72	0.59	Nonsyn	193G > A	Glu65Lys	fig|727.3669.peg.1822	Lipopolysaccharide cholinephosphotransferase LicD1 (EC 2.7.8.-)	missense_variant	MODERATE
727.3669.con.0028	266	769.25	71	0.59	Nonsyn	182A > C	Lys61Thr	fig|727.3669.peg.1822	Lipopolysaccharide cholinephosphotransferase LicD1 (EC 2.7.8.-)	missense_variant	MODERATE
727.3669.con.0028	274	657.764	67	0.58	Synon	174C > T	Phe58Phe	fig|727.3669.peg.1822	Lipopolysaccharide cholinephosphotransferase LicD1 (EC 2.7.8.-)	synonymous_variant	LOW
727.3669.con.0028	280	553.633	63	0.57	Nonsyn	167_168delCAinsAG	Ala56Glu	fig|727.3669.peg.1822	Lipopolysaccharide cholinephosphotransferase LicD1 (EC 2.7.8.-)	missense_variant	MODERATE
727.3669.con.0028	97	821.251	74	0.62	Synon	351G > A	Thr117Thr	fig|727.3669.peg.1822	Lipopolysaccharide cholinephosphotransferase LicD1 (EC 2.7.8.-)	synonymous_variant	LOW
727.3669.con.0029	309	79.9	65	0.52						intergenic_region	MODIFIER

^*a*^ Expanded descriptions of the column headings: Contig (contig name), Pos (position of the variation), Score (quality score from the variant caller tool), Var_cov (variant coverage [the average read depth of the variant]), Var_frac (variant fraction [the fraction of the variant read depth among all the reads that cover this region]), Type (variant type, synon indicates synonymous mutation and nonsynon indicates nonsynonymous mutation), Ref_nt_pos_change (nucleotide change), Ref_aa_pos_change (amino acid change), Gene_ID (BV-BRC feature (peg) id), Function (function description), snpEff_type (SNP variant type), snpEff_impact (SNP variant impact).

We should note that this patient had atherosclerosis, and this risk factor alone might have predisposed him to hematogenous seeding and subsequent development of the infected aneurysm. With that said, while the impact of the genetic changes in this patient’s isolates remains speculative, they could have contributed to the relapsed infection described in this case. The full spectrum of clinical manifestations for invasive NTHi is likely wide-ranging and has yet to be fully described. While beyond the scope of this case report, there is ongoing surveillance of these strains through the CDC-funded Georgia EIP, which is part of larger systematic efforts to further our understanding of the unique pathogenesis of these clonal isolates. Of note, this case occurred outside the timeframe in which the original 2017–2018 case clusters were identified and underscores the continued emergence of novel disease phenotypes for unusually pathogenic strains of NTHi.
